# The Superconducting Mechanism in BiS_2_-Based Superconductors: A Comprehensive Review with Focus on Point-Contact Spectroscopy

**DOI:** 10.3390/nano14211740

**Published:** 2024-10-30

**Authors:** Paola Romano, Aniello Pelella, Antonio Di Bartolomeo, Filippo Giubileo

**Affiliations:** 1 Dipartimento di Scienze e Tecnologie, Università del Sannio, 82100 Benevento, Italy; promano@unisannio.it; 2 CNR-SPIN Salerno, 84084 Fisciano, Italy; adibartolomeo@unisa.it; 3 Dipartimento di Fisica, Università degli studi di Roma Tor Vergata, via della Ricerca Scientifica 1, 00133 Rome, Italy; apelella@unisa.it; 4 Dipartimento di Fisica ‘E R Caianiello’, Università di Salerno, 84084 Fisciano, Italy

**Keywords:** BiS_2_ superconductors, point-contact spectroscopy, pairing symmetry

## Abstract

The family of BiS_2_-based superconductors has attracted considerable attention since their discovery in 2012 due to the unique structural and electronic properties of these materials. Several experimental and theoretical studies have been performed to explore the basic properties and the underlying mechanism for superconductivity. In this review, we discuss the current understanding of pairing symmetry in BiS_2_-based superconductors and particularly the role of point-contact spectroscopy in unravelling the mechanism underlying the superconducting state. We also review experimental results obtained with different techniques including angle-resolved photoemission spectroscopy, scanning tunnelling spectroscopy, specific heat measurements, and nuclear magnetic resonance spectroscopy. The integration of experimental results and theoretical predictions sheds light on the complex interplay between electronic correlations, spin fluctuations, and Fermi surface topology in determining the coupling mechanism. Finally, we highlight recent advances and future directions in the field of BiS_2_-based superconductors, underlining the potential technological applications.

## 1. Introduction

**Figure 1:**
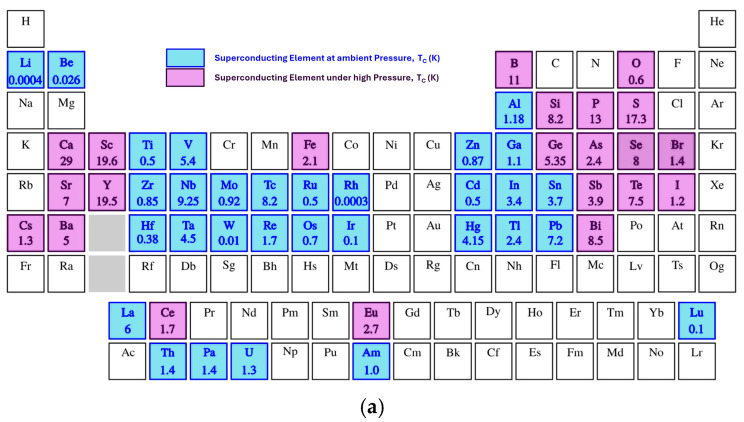

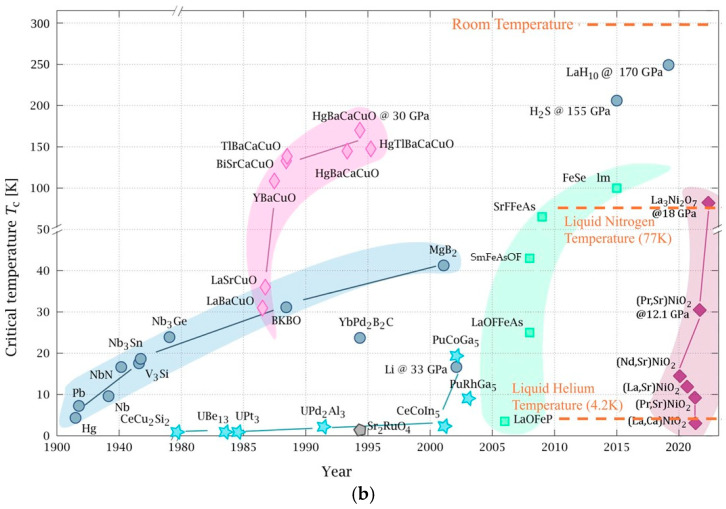
(**a**) Periodic table evidencing superconducting elements either at ambient pressure or under high pressure. (**b**) Time evolution of the superconducting critical temperature (T_C_).

Superconductivity is a macroscopic quantum phenomenon, initially discovered in mercury [1], showing two main properties such as zero electrical resistivity and perfect diamagnetism [2]. Soon after, all elements and simple alloys were investigated to identify among them the superconductors and the corresponding critical temperature (T_C_). Nowadays, 31 elements are known to be superconducting at ambient pressure (with T_C_ in the range from 0.0003 K for Rh to 9.25 K for Nb) [3]. This number increases if considering the characterization under high pressures [4] (see Figure 1a). Similarly, a large number of binary alloys and compounds were found to become superconducting, including compounds in which both elements were non-superconducting, such as Au_2_Bi [5]. The microscopic mechanism of “conventional” superconductivity was explained in 1957 by Bardeen, Cooper, and Schrieffer [6] in their BCS theory with the introduction of “effective” attraction between the electrons at low temperature. The pairing of electrons is phonon-mediated, causing the formation of Cooper pairs that form a Bose-Einstein condensate in the superconducting state. Due to their properties, superconductors can be exploited for several applications, from high efficiency power devices to magnetic energy storage, from power transmission lines to thermonuclear fusion [7]. The main characteristics relevant for such applications are the high-power density and low losses, in order to develop compact, low-consumption, and high-current devices [8]. Clearly, a larger applicability of superconductors aims at as high as possible a critical temperature to reduce the cooling costs. For this reason, the scientific community has continuously searched for new superconducting materials characterized by higher critical temperatures. The first milestone can be identified with the announcement of superconductivity at 30 K in the layered cuprate LaBa_2_CuO_4−x_ by Bednorz and Muller [9], followed soon after (at the beginning of 1987) by the report of a T_C_ as high as 92 K for the YBa_2_Cu_3_O_x_ compound [10], thus opening the field to superconducting applications at temperatures above the boiling point of liquid nitrogen (77 K). Several compounds in the family of cuprates (either hole-doped or electron-doped) have been identified with T_C_ up to 130 K in Hg_2_Ba_2_Ca_2_Cu_3_O_x_. They represent the first class of “unconventional” superconductors, i.e., the superconducting properties being not explained within the BCS theory. The main difficultly in establishing the unconventional character of the cuprates was related to the complex interactions involving electrons, phonons, magnetic fluctuations, and impurities [11]. Indeed, cuprate parent compounds are antiferromagnetic Mott-insulators, and superconductivity appears after carrier doping [12]. The main consequence is that the spin-singlet pairing state with s-wave spatial component assumed in conventional superconductors by the BCS theory does not apply to cuprates, which instead are characterized by a spin-singlet d-wave pairing symmetry [13]. Further advances are represented by the discovery of new unconventional superconductors such as the chiral spin-triplet Sr_2_RuO_4_ [14], the two-band superconductor MgB_2_ [15,16,17,18,19,20,21], the iron-pnictide superconductors with the so-called s±,++ pairing symmetry [22], where the hole and electron pockets develop distinct s-wave gaps having an intrinsic phase difference of 0(s++) or π(s±), filled skutterudite [23], and layered BiS_2_-based compounds [24].

The discovery of superconducting materials that have very high critical temperatures when subjected to extremely high pressures is very recent [25]. It has been reported that hydrogen sulfide (H_2_S) at pressures above 150 GPa has a superconducting transition with T_C_ = 203 K [26]. Similarly, hydrogen-rich materials under extreme compression have shown superconducting behavior, such as lanthanum hydride (LaH_10_), with T_C_ > 250 K [27]. Figure 1b shows the evolution with time of the T_C_ values for the various superconducting materials at ambient pressure.

Finally, the experimental observation of superconductivity with T_C_ in the range 9–15 K has been reported in 2019 for hole-doped infinite-layer nickelate Nd_1−x_Sr_x_NiO_2_ thin films [28]. Several infinite-layer nickelates, chemical formula A_1−x_B_x_NiO_2_, obtained by different combinations of rare earths and alkaline-earths (A  = La, Nd, Pr, and B  =  Sr, Ca), have been already investigated showing some similarities with cuprates [29,30,31,32]. The highest critical temperature T_C_ = 78 K has been reported in 2023 for the La_3_Ni_2_O_7_ under high pressure [33].

The discovery of new superconductors has been always followed by intense research activity to solve the question about the pairing mechanism responsible for the formation of the superconducting state and the symmetry of the superconducting order parameter (OP) [34,35,36,37,38,39,40].

Among the newly discovered superconducting materials, we will focus on the members of the BiS_2_ family, which have attracted considerable attention in recent years due to their intriguing properties and potential technological applications.

**Figure 2:**
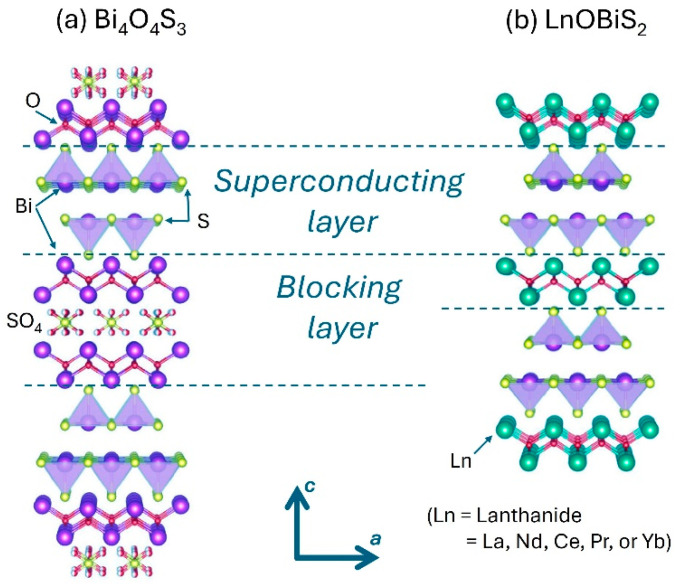
Crystal structure of two BiS_2_-based superconducting materials with indication of superconducting layer and blocking layer: (**a**) Bi_4_O_4_S_3_ and (**b**) LnOBiS_2_.

The first compound of the BiS_2_ family to be reported as a superconductor with T_C_ = 8.6 K was the Bi_4_O_4_S_3_ [41,42], immediately attracting the interest of the whole scientific community. This material has a layered crystal structure, like high-T_C_ cuprates and iron-based superconductors, with superconducting BiS_2_ layers and Bi_4_O_4_(SO_4_)_1−x_ layers as spacers. Soon after, superconductivity was also reported for the compound LaO_0.5_F_0.5_BiS_2_ [43], obtained by modifying the blocking layer in Bi_4_O_4_S_3_. As for Bi_4_O_4_S_3_, LaO_0.5_F_0.5_BiS_2_ is also composed of a stacking of BiS_2_ layers and blocking layers. By modifying the structure and the chemical composition at the blocking layers (by partially substituting O by F), electron carriers are generated, promoting the formation of the superconducting state. Since then, superconductivity has been extensively investigated in several related materials with the formula LnO_1−x_F_x_BiS_2_ (Ln is lanthanide, Ln = La, Nd, Ce, Pr, and Yb) [24,44,45,46]. The schematic of the crystal structure for Bi_4_O_4_S_3_ and for LnOBiS_2_ is reported in Figure 2. Moreover, without introducing F, it has also been shown that electron doping can be increased in the LaOBiS_2_ compound by substituting the trivalent La^3+^ with tetravalent Zr^4+^, Ti^4+^, Th^4+^, and Hf^4+^ [47]. Successively, superconducting compounds based on BiSe_2_ layers have been also reported (as for LaO_0.5_F_0.5_BiSe_2_ with T_C_ = 2.6 K), in which sulfur is replaced by isovalent selenium [48,49]. In Table 1, we summarize the main superconducting compounds of the BiS_2_ family, listed with their critical temperature.

In this review, we summarize the main results obtained on layered BiS_2_-based superconductors related to the pairing symmetry of the superconducting order parameter, with focus on the point-contact spectroscopy (PCS) results. Understanding the symmetry of the superconducting OP provides crucial information about the nature of the superconducting state, offering important insights for future research efforts towards technological advances.

The PCS technique holds significant importance in investigating the symmetry of the order parameter in superconducting materials [90]. The main advantage of PCS is its sensitivity to the superconducting energy gap, enabling precise determination of the pairing symmetry. By measuring the conductance spectra as a function of bias voltage and temperature, PCS can distinguish between different symmetry classes of superconductors, including s-wave, d-wave, or unconventional pairing states. Additionally, PCS offers versatility in probing various regions of the superconductor, providing insights into the spatial variation of the OP, which is crucial for understanding complex superconducting systems like layered BiS_2_ compounds.

This review is organized as follows: In Section 2 we introduce the PCS technique. Section 3 is devoted to summarizing the theoretical background to analyze the PCS experimental results. Section 4 reviews the main results on the pairing symmetry of the BiS_2_-based superconductors.

## 2. The Role of Point-Contact Spectroscopy

PCS has been immediately identified as a powerful tool to investigate the scattering of electrons by elementary excitation in metals, such as phonons [91], since its introduction by Yanson [92]. It has been largely applied in the field of superconductivity because the Andreev reflection (AR) phenomenon allows PCS to provide direct information on the amplitude and symmetry of the superconducting order parameter. When looking at the AR, the technique is often named PCAR (Point-Contact Andreev Reflection spectroscopy). Consequently, in the literature, a great number of reports about conventional and unconventional superconductors are available, investigating the amplitude and the symmetry of the superconducting gap.

### 2.1. PCS Setup Configurations

**Figure 3:**
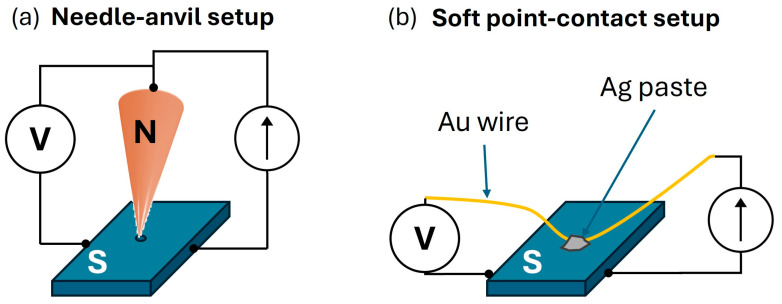
Schematic of two different Point-Contact Spectroscopy setup configurations. (**a**) Needle-anvil setup is realized with a metallic (N) tip pressed onto the superconducting (S) sample surface. (**b**) Soft point-contact setup is realized with a small spot of silver paste fixing a gold wire on the sample surface.

Experimentally, the technique can be easily implemented by pushing a metal tip (N) into contact with a superconducting sample (S), originating a point-contact junction (PCJ) with a small contact area (with respect to the mean free path of the electrons). This setup is commonly referred to as “needle–anvil” configuration and it is equipped with an electrochemically or mechanically sharpened metallic tip (typical curvature radius is of the order of several micrometers) that can be gently pressed on sample surface (a schematic is shown in Figure 3a). The main advantage of this technique is related to the possibility of finely tuning the tip pressure on the surface, modifying the resistance of the PCJ. Moreover, the setup allows the experiment to be repeated in many different areas of the same sample, investigating the sample homogeneity as well.

On the other hand, tip pressure can cause deformations at the interface with the creation of several parallel contacts. This is not a limitation to performing the spectroscopic analysis if the sample is homogeneous on a length scale larger than tip diameter [93,94,95]. The main drawback of the needle–anvil setup is related to the junction stability, affected by thermal and mechanical variations, becoming very relevant if studying very small samples.

A different approach to realize the PCJ is the so-called ‘soft’ point-contact technique [96,97] in which the metallic tip pressed on the sample surface is substituted by a small drop of Ag paste (or Indium flake) fixing a metallic (typically gold) wire to be used as electrical leads (see Figure 3b). The main advantage of this setup is the high mechanical and thermal stability of the junction. It has been demonstrated that, despite the relative large area of the Ag island used to create the contact, the real contact is formed by few to several parallel nanometric constrictions, thus allowing spectroscopic insights to be obtained [96].

### 2.2. Conduction Regimes in PCJ

Three possible conduction regimes can take place in a PCJ: (I) ballistic regime, (II) thermal regime, and (III) intermediate regime. The different regimes depend on the contact size. For this reason, the reference parameter is the Knudsen ratio K, expressed in terms of the electron mean free path l and the contact dimension a, according to the definition K=l/a (considering a circular contact of radius a). The ballistic regime corresponds to the situation in which the contact size is very small with respect to the mean free path (a≪l, i.e., K≫1) and an electron can be accelerated through the PCJ by a voltage V without scattering and gaining an energy eV. According to the Sharvin formula, the contact resistance (known as Sharvin resistance) in the ballistic regime can be written $R_{PCJ}^{Sharvin}=4\rho l/3\pi a^{2}$, where we indicate with ρ the resistivity of the sample [98]. The opposite condition of large contact (a≫l, i.e., K≪1) corresponds to the thermal regime (also known as the Maxwell regime). In this case, electrons cannot avoid inelastic scattering in the contact region, and the conduction through the junction is dissipative, causing contact heating. In this regime, the contact resistance is expressed by the Maxwell formula $R_{PCJ}^{Maxwell}=\rho /2a$ [99]. The intermediate regime corresponds to the case a<l, in which the contact size is smaller than the inelastic scattering length. In this situation, contact heating is negligible, and energy resolved spectroscopy can be performed. The contact resistance can be obtained through the Wexler formula that includes both the Maxwell and the Sharvin resistance as $R_{PCJ}^{Wexler}=f\left(K\right)\cdot \rho /2a+4\rho l/3\pi a^{2}$, where $f\left(K\right)$ is a function of the Knudsen ratio and it has values around one [100].

### 2.3. Andreev Reflection

**Figure 4:**
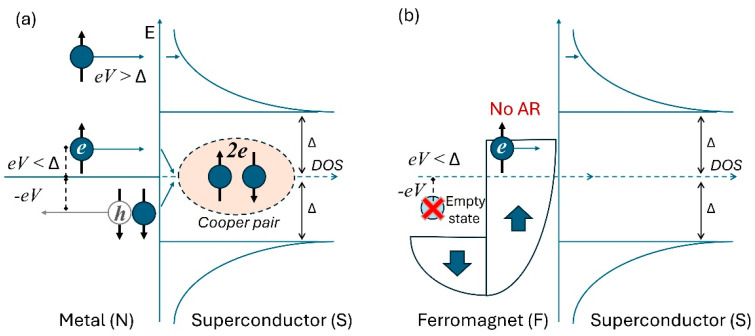
(**a**) Schematic of the Andreev reflection at the N/S interface. (**b**) Suppression of the AR process at the F/S interface.

When characterizing a PCJ formed between a metallic tip and a superconductor sample, an electron coming from the N electrode can reach the N/S interface with an energy lower than the superconducting energy gap (∆), and, consequently, cannot pass through, having no available states on the other side. However, the transport current can be allowed by the Andreev reflections, i.e., the phenomenon for which such electron with energy E<∆ can form a Cooper pair with another electron to enter in the superconducting electrode, while a hole, with opposite momentum with respect to the incident electron, is reflected in N [101] (see Figure 4a). A single Andreev reflection process causes a charge transfer through the N/S interface of a total charge 2e. When the interface is highly transparent (low barrier), almost all incident electrons with E<∆ undergo to Andreev reflection, so that the overall junction conductance results to be twice the normal conductance. Consequently, PCS becomes an obvious method to directly measure the amplitude of the superconducting energy gap for a material under investigation. Andreev reflections are theoretically described by the solution of the Bogoliubov–de Gennes equations near the N/S interface [102,103]. It is worth noting that ARs are not limited at the interface but can extend for a length ξ (which corresponds to the length scale over which the presence of the N electrode reduces the superconductivity in S due to the proximity effect). Only when the contact size is lower than ξ, can the proximity effect be neglected.

The great applicability of the PCAR technique is demonstrated by the huge numbers of experiments that have been reported on several superconducting materials such as conventional BCS superconductors [91,104,105,106], electron-doped and hole-doped cuprates (hig T_C_ superconductors) [37,107,108,109,110,111,112], rutheno-cuprates [39,113,114], MgB_2_ [17,96,97,115,116,117,118,119,120,121,122], filled skutterudite [123,124], iron pnictides [125,126,127,128,129,130,131], heavy fermions [132,133,134,135,136,137,138], non-centrosymmetric [139,140,141], and topological superconductors [142,143].

Interestingly, the AR process can also be exploited to measure the spin polarization (P) in PCS experiments. If the metal electrode is ferromagnetic (F), the probability that AR takes places at the interface F/S is reduced because the spin-up and spin-down bands in F are different (see Figure 4b). Indeed, the AR near the Fermi level preserves energy and momentum but not spin: the incoming electron and the reflected hole have opposite spin. For the N/S interface, this is not irrelevant, because of the spin rotation symmetry. Differently, for F/S interfaces, the spin flipping is crucial: in fully spin-polarized metals all carriers have the same spin, and the AR is completely suppressed.

Indeed, this technique has been exploited in several experiments to characterize ferromagnetic metals, such as Fe, Ni, Co [144,145,146,147], half metals [148,149], ferromagnetic alloys [144,150], manganites [144,151,152], and ruthenates [153,154]. Also, it has been reported that a gold tip on the PdNi/Nb bilayer enables the resonant proximity effect, providing conductance features very sensitive to the local ferromagnetic properties and allowing an accurate measurement of polarization and thickness of the ferromagnetic layer by PCS [155,156,157].

## 3. Theoretical Background

In a PC geometry, the junction can be easily modified by pushing/retracting a metallic tip. Typically, larger pressure on the surface of the superconducting sample corresponds to higher barrier transparency at the N/S interface. Consequently, by controlling the tip it is possible to modify the conduction regime from pure tunnelling (i.e., high potential barrier corresponding to low transparency of the interface) to the case of direct transparent contact (characterized by low barrier at the interface). Such tunability of the conduction regime has been well described for conventional superconductors (s-wave symmetry of the order parameter) within the Blonder–Tinkham–Klapwijk (BTK) theory [158], where a dimensionless parameter Z is used to model the height of a delta function representing the potential barrier within the Bogoliubov–De Gennes equation. In this representation, Z = 0 corresponds to a perfectly transparent interface, while, for increasing Z, the transparency is reduced towards a complete tunnelling regime. Of course, intermediate regimes can realize intermediate barrier strengths, so that both quasiparticle tunnelling and Andreev reflection processes contribute to the overall conduction through the interface. To calculate the differential conductance, $G_{NS}=dI/dV$, the BTK theory considers both the normal reflection probability, B(E), and the Andreev reflection probability, A(E), for an electron approaching the N/S interface (from the N side):

(1) $$G_{NS}=G_{NN}\int_{-\infty }^{+\infty }\left[1+A\left(E\right)-B\left(E\right)\right]\frac{df\left(E+eV\right)}{d\left(eV\right)}dE$$

where $G_{NN}$ is the conductance in the normal state that can be expressed in terms of the barrier strength as $G_{NN}={\left(1+{\left(Z/2\right)}^{2}\right)}^{-1}$, V is the applied potential, and f(E) is the Fermi function.

**Figure 5:**
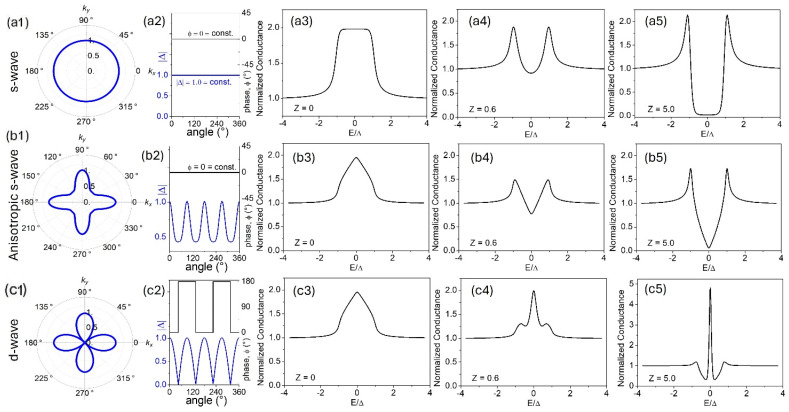
Comparison of OP symmetries: s-wave (first row), anisotropic s-wave (second row), and d-wave (third row). First column (**a1**,**b1**,**c1**) shows the polar plots (**a2**,**b2**,**c2**) of the OP in the momentum plane, modulus, and phase of the OP; (**a3**,**b3**,**c3**) conductance spectra calculated for Z = 0; (**a4**,**b4**,**c4**) conductance spectra calculated for Z = 0.6; (**a5**,**b5**,**c5**) conductance spectra calculated for Z = 5.0. For d-wave spectra, numerical simulation has been performed for α = π/8.

From Equation (1), it is clear that an electron reflected at the interface causes a reduction of the current through the interface. At the same time, if the electron undergoes the Andreev reflection, it causes an increase in the current because it matches another electron to form a Cooper pair that enters in the superconducting side of the junction (as depicted in Figure 4a). In the presence of a completely transparent barrier (Z = 0), the transport current is dominated by the Andreev processes, and, consequently, the differential conductance is twice the normal conductance, i.e., $G_{NS}/G_{NN}=2$ (Figure 5(a1–a3)).

By increasing Z, the Andreev reflections are partially suppressed and the conductance spectra for Z > 1 tend to the case of N/I/S tunnel junctions (with I representing an insulating barrier) showing peaks at eV=±Δ (Figure 5(a4,a5)).

Kashiwaya and Tanaka extended the BTK model considering different symmetries of the superconducting order parameter. Indeed, for a d-wave superconductor, the electron-like and hole-like quasiparticles, incident at the N/S interface, experience different signs of the OP, with formation of Andreev Bound States at the Fermi level along the nodal directions. The Andreev Bound States modify the transport current, and, consequently, the expression of the differential conductance, which can be written:

(2) $${\tilde{G}}_{NS}\left(V\right)=\frac{1}{\int_{-\infty }^{+\infty }dE\left[-\frac{df(E+eV)}{d(eV)}\right]\int_{-\frac{\pi }{\;2}}^{\frac{\pi }{\;2}}d\varphi \sigma_{N}\left(E\right)cos\varphi }\int_{-\infty }^{+\infty }dE\int_{-\frac{\pi }{\;2}}^{\frac{\pi }{\;2}}d\varphi \sigma \left(E,\varphi \right)cos\varphi \left[-\frac{df(E+eV)}{d(eV)}\right]$$

with

$$\sigma \left(E,\varphi \right)=\sigma_{N}\left(\varphi \right)\frac{\left(1+\sigma_{N}\left(\varphi \right)\right)\Gamma_{+}^{2}+\left(\sigma_{N}\left(\varphi \right)-1\right){\left(\Gamma_{+}\Gamma_{-}\right)}^{2}}{{\left(1+\left(\sigma_{N}\left(\varphi \right)-1\right)\Gamma_{+}\Gamma_{-}\right)}^{2}}$$

in which

$$\sigma_{N}\left(\varphi \right)={\left(1+{\tilde{Z}\left(\varphi \right)}^{2}\right)}^{-1},\tilde{Z}\left(\varphi \right)=Z\cos\left(\varphi \right),\;\Gamma_{\pm }={\left(\Delta_{\pm }\right)}^{-1}\left(E-\sqrt{E^{2}-{\Delta_{\pm }}^{2}}\right)$$

and $\Delta_{\pm }=\Delta \;\cos[2(\alpha \mp \varphi )].$

At a given energy E, the transport current depends both on the incident angle φ of the electrons at the N/S interface as well as on the orientation angle α, that is, the angle between the a-axis of the superconducting OP and the *x*-axis of the crystal structure. When applying Equation (2) to PC experiments, there is no preferential direction of the quasiparticle injection angle φ from N into S, so the transport current results by an integration over all directions inside a semisphere weighted by the scattering probability term in the expression for the current.

For anisotropic s-wave superconductors, the amplitude of the OP varies in the k-space, while its phase remains constant, and the extended model can be simplified considering that $\Delta_{+}=\Delta_{-}=\Delta \cos\left[2\left(\alpha -\varphi \right)\right]$ (Figure 5(b1,b2)).

In this case, in the limit of the transparent barrier (Z→0), an increase in the conductance for E<Δ with a triangular profile is found with maximum amplitude $G_{NS}/G_{NN}=2$ at zero bias (Figure 5(b3)). On the other hand, for higher Z, we obtain tunnelling conductance spectra that show the characteristic “V”-shaped profile (Figure 5(b4,b5)) in comparison to the classical “U”-shaped structure found for an isotropic s-wave OP. We notice that for anisotropic s-wave superconductors, the conductance curves are quite insensitive to α and a zero bias conductance peak (ZBCP) is obtained only for low barriers (small Z).

In the case of a d-wave symmetry (Figure 5(c1,c2)), for Z→0, the conductance curves at low temperatures show again a triangular structure centered at eV = 0 (Figure 5(c3)), quite insensitive to variations of α with maximum amplitude $G_{NS}/G_{NN}=2$. However, for higher barriers (Figure 5(c4,c5)), the conductance characteristics show dramatic changes as a function of α. In particular, as soon as α ≠0, the presence of Andreev Bound States at the Fermi level produces strong effects more evident along the nodal direction (α=π/4) for which $G_{NS}/G_{NN}>2$ is found.

## 4. Results on BiS_2_-Based Superconductors

Layered materials show interesting electronic and magnetic properties, thanks to their two-dimensional crystal structure and electronic states. In particular, exotic superconductivity seems to prefer a layered crystal structure. For example, high T_C_ superconductivity is observed in layered materials, such as cuprates [9,10,159,160], Fe-based [22,161,162,163,164,165,166,167], and MgB_2_ [15,168] materials. Among the layered superconductors, the chalcogenides constitute one of the most interesting groups, due to the observation of exotic superconductivity. Sulfur (S), selenium (Se), and tellurium (Te) are categorized as chalcogens [169].

The results are controversial as to the possible mechanism at the basis of superconductivity in BiCh_2_-based (Ch:S,Se) materials. The gap symmetry of layered superconductors is still an open issue. Both theoretical and experimental investigations have been performed, but, to our knowledge, not many works focus on the direct measurement of size and symmetry of the order parameter.

Early theoretical models [170,171] suggested a conventional strong-coupling mechanism, while subsequent works [40] proposed weak-coupling electron–phonon mechanisms.

The carriers’ doping effect has been investigated by means of computational methods. Theoretical simulations based on density functional theory have been performed by Al-Amer et al. [172] simulating the electronic structure characteristics of Sr_1−x_Hf_x_FBiS_2_, with x from 0 to 1. The evaluated band gap of the parent compound (SrFBiS_2_) is roughly about 0.88 eV, which vanishes under the substitutional impurity impact of tetravalent (Hf + 4). However, a metallic character occurs when Hf substitutes Sr in the SrFBiS_2_ parent compound. The optical characteristics have also been investigated; the optical anisotropy in the absorption spectra has been found to be well marked in the parent material, while it diminishes through the substitutional Hf impurity effect. From the results, the author suggests that the SrFBiS_2_ parent compound could be a good candidate for optical communications and laser devices.

The phonon dispersion of LaBiS_2_O_0.5_F_0.5_ has been investigated by first-principles calculations and inelastic X-ray scattering experiments by Tamatskuri et al. [173]. The results show that the phonon mode corresponding to the transverse-type lattice modulation is unstable, suggesting that phonon softening may originate from the Fermi surface nesting. The authors suggest two possibilities for the transverse lattice modulation in LaBiS_2_O_0.5_F_0.5_: the order–disorder-type structural transition and the displacive structural transition with an overdamped mode, for both of which the local structure distortion or the short-range correlation within the BiS_2_ plane would be essential.

On the experimental side, several techniques usually used for the investigation of superconductivity have been applied to study the electronic structure of BiS_2_-based superconductors.

**Figure 6:**
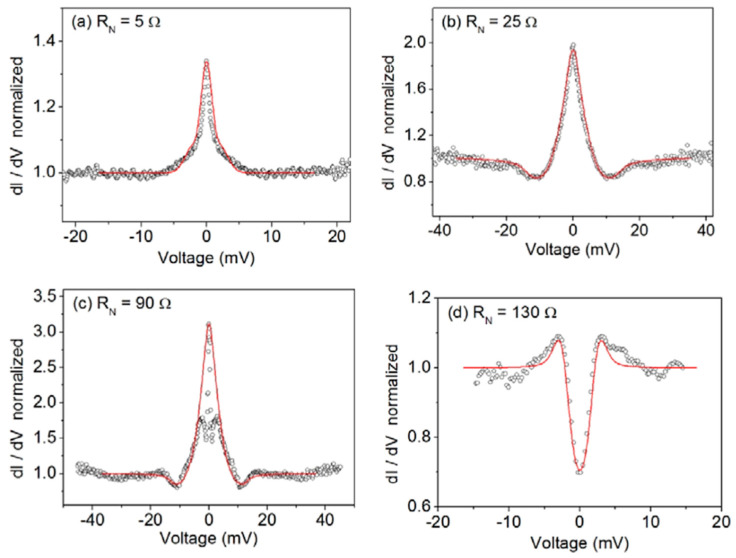
Differential conductance vs. voltage for different resistance contacts made by point-contact spectroscopy on LaO_0.5_F_0.5_BiS_2_, reproduced with permission from Ref. [35].

Unconventional pairing in LaO_0.5_F_0.5_BiS_2_ has been inferred by means of point-contact spectroscopy [35] by pushing a gold tip onto the surface of polycrystalline samples. Different contacts have been realized, with different normal resistances. The differential conductance vs. voltage measured at low temperature appears in several types (see Figure 6).

**Figure 7:**
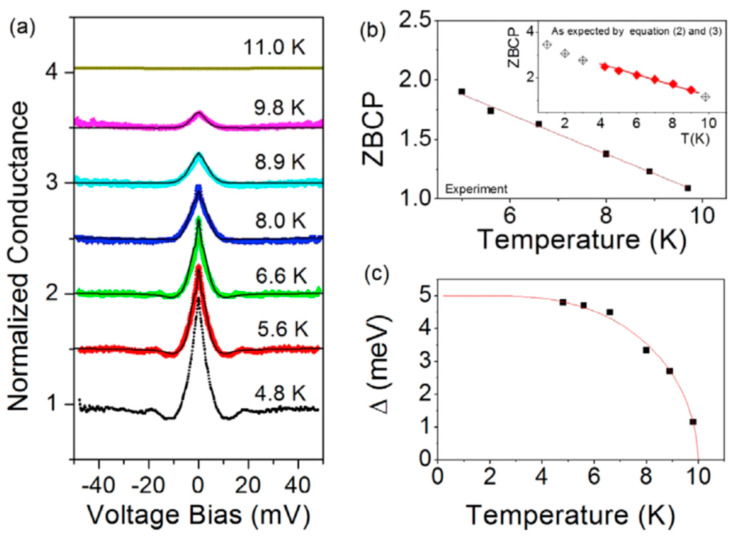
(**a**) Normalized differential conductance measured at different temperatures for a low resistance contact on LaO_0.5_F_0.5_BiS_2_. (**b**) ZBCP amplitude as a function of temperature. (**c**) Energy gap value obtained by the fits as a function of temperature. All the data are reproduced with permission from Ref. [35].

Although the data seem to be very different from each other, a best fit (red curves) can be obtained by using the BTK model with a d-wave order parameter symmetry. Different Z values have been used; a smearing parameter has been introduced to consider the finite lifetime of the quasiparticles and pair-breaking effects. Although the fits appear to be good, the resulting energy gap values are similar and reasonable only for the curves a and d, i.e., around 4.2 meV. For the other two junctions, b and c, the values used in the fits are much higher, around 13–14 meV. However, the possible formation of intergrain Josephson junctions (JJ) in series with the point contact has been considered. The introduction of JJ also introduces two more parameters into the fitting procedure, the critical current I_JJ_ and the resistance R_JJ_. When added to the point-contact resistance, R_JJ_ gives the normal resistance R_N_. For low R_N_, like, for instance, those of figure a and b, there is no evidence of a contribution from JJ. Considering the JJ contribution, the energy gap fitting parameter for the curves b and c becomes 4.8 meV, consistent with curves a and d. On the other hand, a ZBCP is observed for the low resistance contacts, related to Andreev reflections in the low transparency NS contact. The ZBCP disappears above T_C_, as shown in Figure 7, where the amplitude of the ZBCP for the 25 Ω contact has been extracted from the conductance measured at different temperatures up to 11 K. The energy gap value obtained by the fittings is also reported and shows the expected decreasing superconducting behavior.

**Figure 8:**
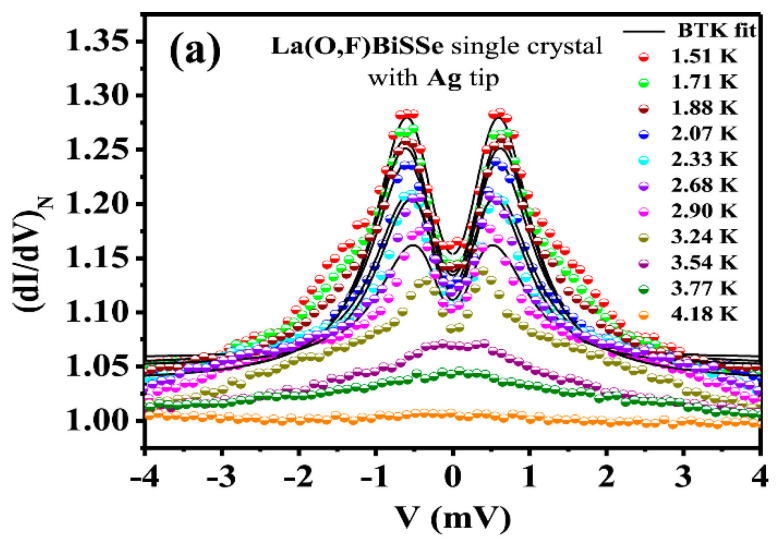

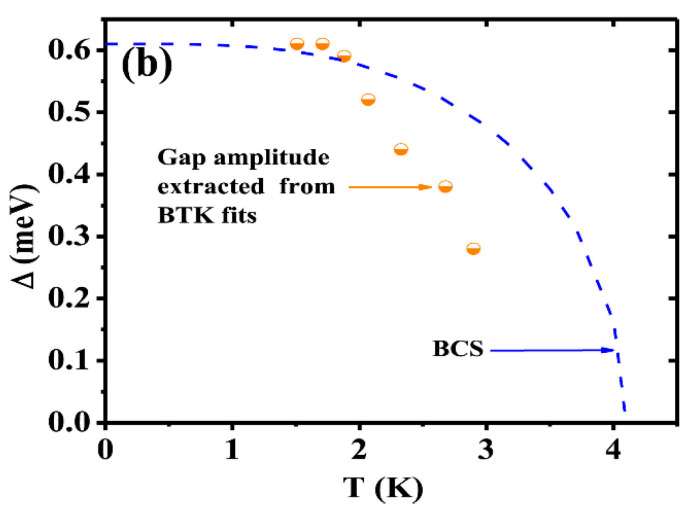
(**a**) Normalized differential conductance measured on single crystals of LaOFBiSSe by means of PCS at different temperatures. (**b**) Gap amplitude as a function of temperature. All the data are reproduced with permission from Ref. [174].

Aslam et al. [174] performed PCS using a metallic tip of Ag on single crystals of LaOFBiSSe. A conventional four-contacts configuration was used for forming ballistic point contacts, and the differential conductance was directly recorded by means of a lock-in modulation technique. Low temperature data show a two-peak feature conductance (see Figure 8).

The BTK theory with a broadening parameter was used to fit the data, and a value of the gap of 0.61 meV was found at the lowest temperature of 1.51 K. While the lower part of the spectra was well fitted, the higher biases data show a significative deviation from the BTK behavior. Such a difference could be due to a multigap as observed in MgB_2_, although as the temperature is raised, but still below the T_C_, the additional spectra features disappear and the BTK behavior is restored. Instead, in MgB_2_, the features associated with the two gaps survive up to T_C_ and, for this reason, are connected to the superconducting state. Furthermore, the temperature dependence of the energy gap decreases in a way different from the one expected from the BCS theory, suggesting an unconventional nature of the superconductivity in this material. A magnetic field was also applied along the *c*-axis of the crystal, as well as in the *ab*-plane. The additional “higher gap” feature observed at zero field disappears at finite field, and the spectra are well fitted with the BTK at any value of the applied field, either in the *c* direction and in the *ab* plane. The results, however, show an evident anisotropy in the superconducting properties. The energy gap amplitude indeed decreases faster with the magnetic field applied along the *c*-direction than in the *ab*-plane. The anisotropic properties were also investigated by means of measurements made at different field angles (orientation θ ϕ of the applied magnetic field) for fixed magnetic field values, in order to obtain both polar (ϕ = 0 and changing θ) and azimuthal (θ = 0 and changing ϕ) dependence. From the observed behavior, the authors conclude that the superconducting gap has a complex symmetry with different anisotropy factors in different planes.

**Figure 9:**
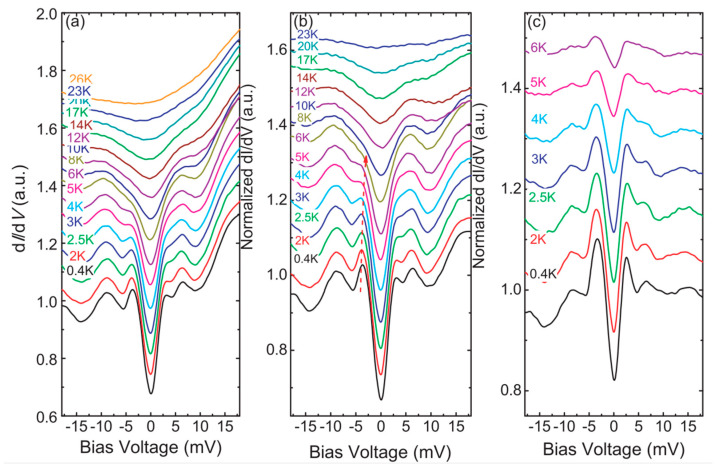
STM spectra obtained on single crystals of NdO_0.5_F_0.5_BiS_2_. Data reproduced with permission from Ref. [176]. (**a**) Temperature evolution of STS spectra for temperature range from 0.4 K to 26 K. (**b**) The STS spectra are normalized by the one measured at 26 K. A dashed red line highlights the superconducting coherence peaks at around 3.5 meV. (**c**) The STS spectra are normalized by the one measured at 8 K.

Tunnelling spectra were also recorded by Liu et al. [175]. Single crystals of NdO_0.5_F_0.5_BiS_2_ grown by the flux method were characterized by means of resistivity measurements that showed a giant superconducting fluctuation effect above T_C_. This effect was also confirmed by Nernst measurements. On one of these samples, Scanning Tunnelling Spectroscopy (STM) measurements were performed. The low temperature spectra exhibit what the authors describe as a two-gap behavior, a smaller gap $\Delta_{1}\approx 3.5\;meV$ and a larger gap $\Delta_{2}\approx 7.5\;meV$ (see Figure 9).

The smaller gap disappears above 6 K, and, for this reason, it has been related to the bulk superconducting transition of 5 K, with a corresponding ratio $\frac{2\Delta_{1}}{k_{B}T_{c}}=5.92$. On the other hand, the larger gap, which looks like a hump feature, persists up to 26 K. Since Liu et al. do not fit their data to any model, the position of the mentioned structure is just what observed in the raw data, and it could come from other effects not related to the energy gap. Dip–hump features have for instance been observed in the past in the tunnelling spectra of high-T_C_ superconductors. In Bi_2_Sr_2_CaCu_2_O_8+δ_ samples, e.g., it has been argued that the observed dip–hump structure may arise from state-conserving deviations in the superconducting density of states, e.g., from the strong-coupling effect [177].

**Figure 10:**
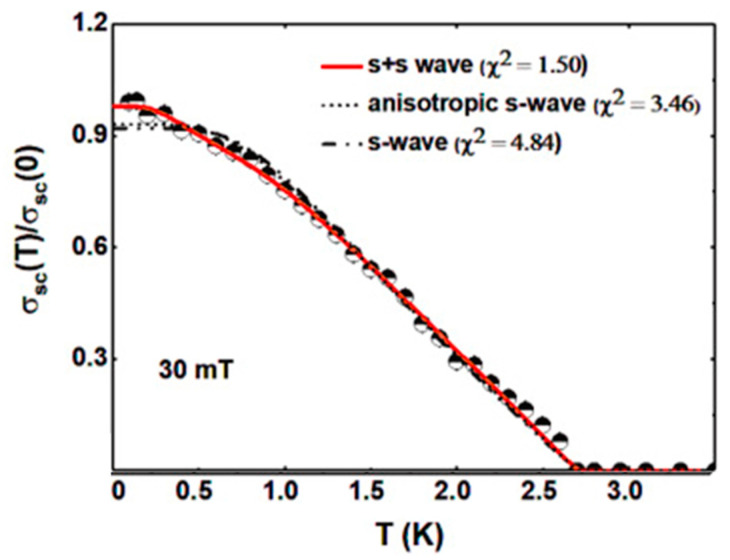
Superconducting muon relaxation rate of La_0.7_Ce_0.3_OBiSSe reproduced with permission from Ref. [178].

Multigap superconductivity has also been inferred in La_0.7_Ce_0.3_OBiSSe, through transverse field (TF) muon spin rotation measurement, magnetization, resistivity, and zero field (ZF) muon spin relaxation measurements by Bhattacharyya et al. [178]. The total Gaussian muon de-polarization rate σ, i.e., the total sample relaxation rate, contains from both the vortex lattice (σ_sc_) and nuclear dipole moments (σ_nm_), which are assumed to be constant over the entire temperature range. The superconducting contribution to the muon relaxation rate is calculated using [σ_sc_ = (σ^2^ − σ^2^_nm_)^1/2^]. σ_sc_ is directly, in the high Hc_2_ limit, related to the superfluid density, and the temperature dependence of σ_sc_(T)/σ_sc_(0), which is related to energy gap for quasi-particle excitations, was fitted with a single s-wave, anisotropic s-wave, and s+s-wave models. From the fit to the superconducting data, it is clear that the superconducting gap structure is best modeled by an isotropic s + s-wave model compared to a single s-wave model or an anisotropic s-wave model, which is in agreement with the theoretical predictions of BiCh_2_-based superconductors (see Figure 10).

**Figure 11:**
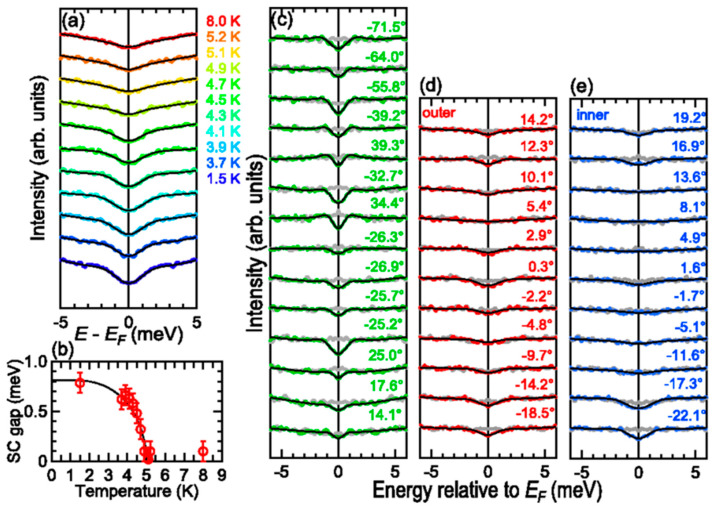
(**a**–**e**): Energy distribution curves obtained by ARPES on NdO_0.71_1F_0.29_BiS_2_ single crystals. (**b**): Energy gap value obtained from the fits of curves (**a**) as a function of temperature. Figure is reproduced with permission from Ref. [180].

Angle-resolved photoemission spectroscopy (ARPES) performed on high-quality NdO_0.54_F_0.46_Bi_0.84_S_1.87_ single crystals with a T_C_ of 4.87 K [85] suggest that the BiS_2_-based superconductors could be conventional BCS superconductors mediated by electron–phonon coupling. The results in Ref. [85] show indeed that the superconductivity can survive for a system with a much smaller Fermi surface volume than that predicted by theories, and that the electron correlation is very weak as well. Therefore, the unconventional quantum-fluctuation-mediated pairing mechanism seems to be not occurring in the BiS_2_-based superconductors. Instead, this compound seems to behave like a multi-band superconductor due to electron–phonon coupling, such as MgB_2_. The electronic structure of LaO_1−x_F_x_BiS_2_ single crystals with nearly optimal doping (x = 0.46) was also measured by ARPES [179]. The observed valence bands and band dispersions near E_F_ show a good agreement with the results of first principles band calculations with spin–orbit coupling. For this reason, the authors suggest a low electron correlation, with a clear influence of the spin–orbit coupling on the electronic structure. A strongly anisotropic superconducting gap was observed in ARPES measurements performed on NdO_0.71_1F_0.29_BiS_2_ single crystals, suggesting that the pairing mechanism is an unconventional one and that the anisotropy can be ascribed to competitive or cooperative multiple paring interactions [180]. The superconducting gap size was obtained by fitting the energy distribution curves to the BCS spectral function [5], and the Δ(T) behavior is shown in Figure 11.

Unconventional superconductivity was also proposed from the study of the evolution of fine electronic states in superconducting LnO_1−x_F_x_BiS_2_ (Ln stays for lanthanoid) [181]. Small elliptic electronic pockets in the superconducting samples have been identified in the low-doping samples, in contrast with previous theoretical scenarios of nesting vectors between the larger starlike electronic pockets around X(π,0).

**Figure 12:**
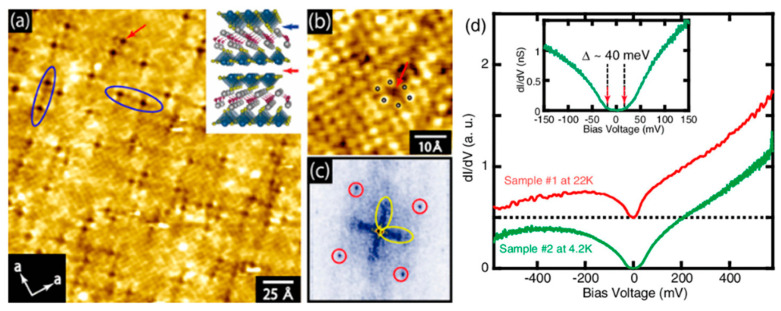
(**a**,**b**): STM images. The inset in (**a**) is a schematic of the crystal structure, with red, gray, yellow, and blue spheres representing Nd, O, S, and Bi atoms, respectively. (**c**): Fourier transform of (**a**). Red lines show the spots corresponding to the atomic array. Yellow lines indicate the tails corresponding to the dark streaks running along the diagonal (110) direction of the unit cell. (**d**): Tunnelling spectra on single crystals of NdO_0.7_F_0.3_BiS_2_. The figure is reproduced with permission from Ref. [182]. © (2014) The Physical Society of Japan.

Scanning tunnelling microscopy (STM) has been applied to single crystals of NdO_0.7_F_0.3_BiS_2_, revealing the existence of a novel electronic structure in the BiS_2_ plane [182]. Tunnelling spectra below and above T_C_ showed a large spectroscopic gap (∼40 meV), inconsistent with the metallic nature demonstrated in bulk measurements. The observed feature is not considered a superconducting gap since it persists well above T_C_ and is not combined with coherence peaks (see Figure 12).

**Figure 13:**
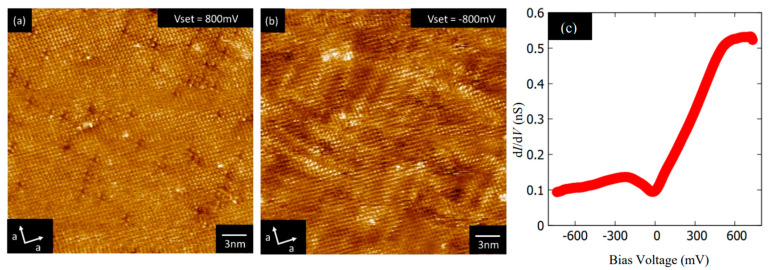
(**a**,**b**): STM images. (**c**): Tunnelling spectra on LaO_0.9_F_0.1_BiSe_2_ single crystals. The figure is reproduced with permission from Ref. [183].

On the other hand, STM on LaO_0.9_F_0.1_BiSe_2_ single crystals showed a finite local density of states at the Fermi energy [183] (see Figure 13) consistent with metallic conductivity revealed in electric resistivity measurements on single crystals [184] and in bulk samples [185].

Phonon-mediated superconductivity for BiS_2_-based materials has also been suggested by Raman scattering experiments [186] and magnetic penetration depth measurements [84].

Thermal conductivity measurements on a high-quality single crystal of NdO_0.71_F_0.29_BiS_2_ down to 100 mK [187] seem to show fully gapped superconductivity with a conventional s-wave superconducting state. To investigate whether electron–phonon interaction is the basis of the pairing mechanism, the isotope effect is one of the most indicated methods routinely used in superconductivity, in agreement with the BCS theory [6]. No isotope effect on T_C_ for LaO_0.6_F_0.4_BiSSe with ^76^Se and ^80^Se was observed [65], suggesting that phonons may not be essential for the pairing in chalcogenides.

**Figure 14:**
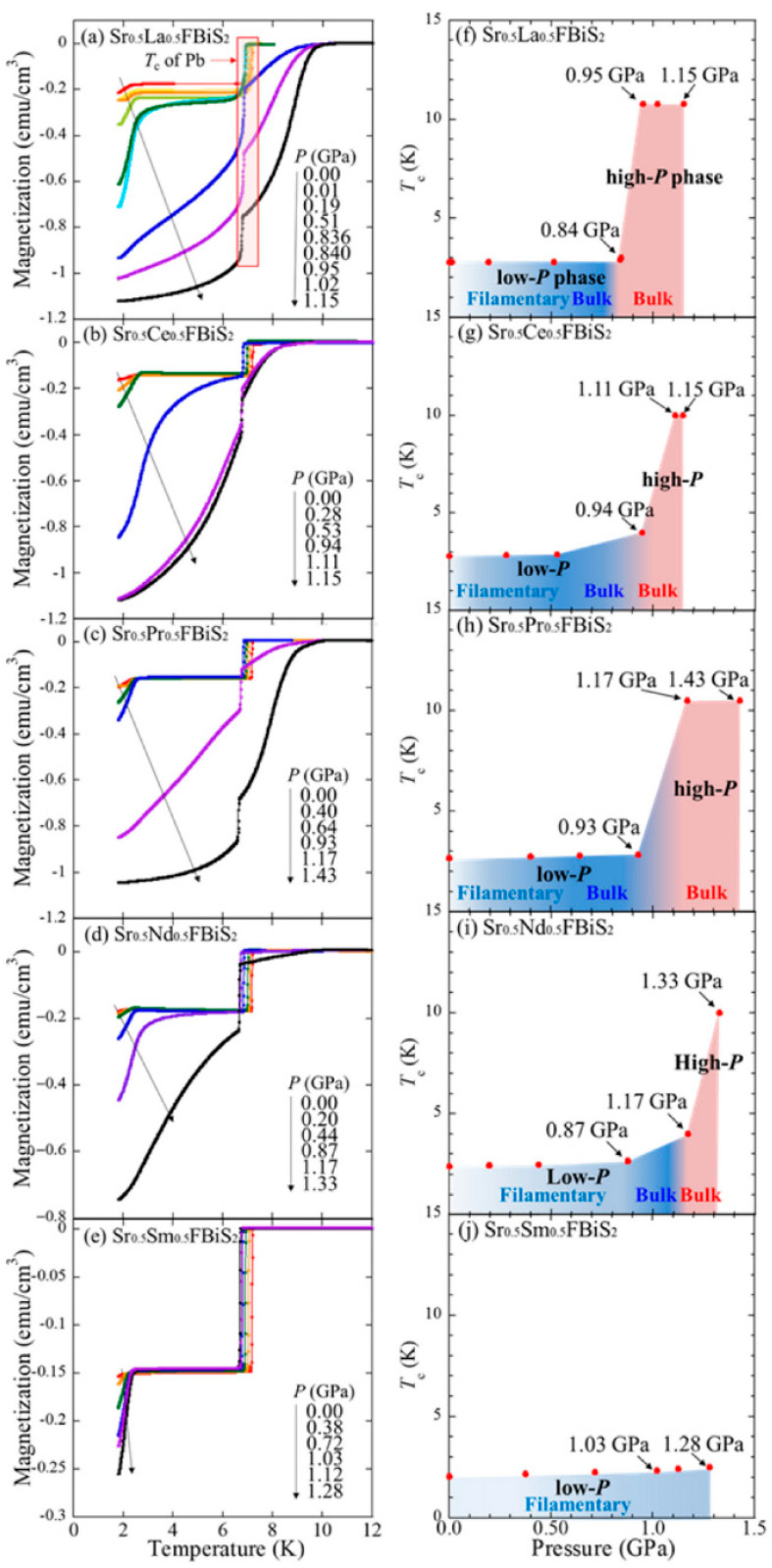
(**a**–**e**): The effects of external pressure on magnetization for Sr_0.5_RE_0.5_FBiS_2_ samples with RE = La, Ce, Pr, and Nd. (**f**–**j**): Pressure dependence of T_c_. This figure is reproduced from Ref. [188] in compliance with CC BY license.

Based on systematic structural analyses, it has been revealed that the in-plane chemical pressure is one of the essential parameters that facilitate the emergence of bulk superconductivity in BiS_2_-based compounds. The relationship between external pressure effects, chemical pressure effects, and the evolution of superconductivity in Sr_0.5_RE_0.5_FBiS_2_ was analyzed by Yamashita et al. [188]. Structure analysis, resistivity, and magnetic susceptibility measurements were studied under ambient and high pressures for Sr_0.5_RE_0.5_FBiS_2_ (RE: La, Ce, Pr, Nd, and Sm). The effects of external pressure on magnetization resulted in abrupt increments in T_C_ up to 10–10.8 K for the samples with RE = La, Ce, Pr, and Nd. The pressure dependence of T_C_ is summarized in Figure 14; the light blue, blue, and pink regions indicate the filamentary superconductivity, bulk superconductivity in the low-P phase, and bulk superconductivity in the high-P phase, respectively.

Tomita et al. [73] showed that T_C_ can be increased by applying pressure in the case of polycrystalline samples. S. Yamamoto et al. [189] studied high-pressure effects on the electrical transport properties of La(O,F)BiS_2_ single crystals, and a discrete enhancement in the T_C_ was observed at 0.9 GPa. The T_C_ values at pressures above 0.9 GPa of a single-crystal sample result slightly lower than those of a polycrystalline sample. A linear decrease in T_C_ with the application of pressure is typically observed in conventional BCS-type superconductors such as MgB_2_ [190]. The applied pressure is expected to reduce the density of states at the Fermi energy N(0) and enhance the phonon frequency ω because of bandwidth broadening and phonon hardening, respectively. A drastic decrease in the electron–phonon coupling constant λ, however, gives a net T_C_ reduction. The transport measurements and Raman studies under high pressures revealed that the discrete enhancement in T_C_ originated from the structural phase transition. A linear reduction in T_C_ and phonon hardening were observed with an increase in the applied pressure on the high-pressure phase of the La(O,F)BiS_2_ single crystal, supporting the phonon-mediated pairing mechanism of superconductivity in La(O,F)BiS_2_.

**Figure 15:**
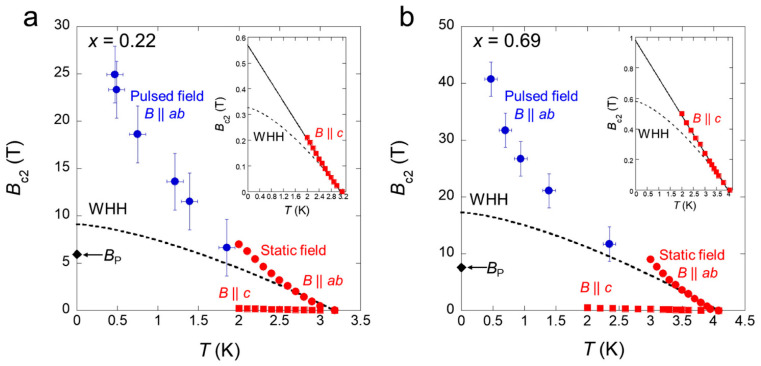
In-plane Bc_2_ in LaO_0.5_F_0.5_BiS_2−x_Se_x_ for (**a**) x = 0.22 and (**b**) x = 0.69. Figure is reproduced from Ref. [191] in compliance with CC BY license.

An unexpected high upper critical field has been observed in dichalcogenides materials. Superconducting states are usually destroyed when a magnetic field is applied to the superconductor, and the maximum field is the upper critical field Bc_2_ in type-II superconductors. Superconductors that yield high Bc_2_ have attracted significant attention. Bc_2_ is determined by two distinct pair-breaking effects: the paramagnetic pair-breaking effect and orbital pair-breaking effect. In 2D superconductors of transition-metal dichalcogenides, the orbital pair-breaking effect is almost quenched, and the paramagnetic pair-breaking effect is predominant, so that the broken in-plane inversion symmetry plays a significant role in superconductivity. The Zeeman-type spin–orbit coupling providing an Ising state causes the enhancement of the Bc_2_. Hoshi et al. [191] have shown that the inversion symmetry is locally broken in the BiCh_2_ layer and this causes extremely high in-plane Bc_2_ in LaO_0.5_F_0.5_BiS_2−x_Se_x_ (x = 0.22 and 0.69). The superconducting states were not completely suppressed by applied fields with strengths up to 55 T (see Figure 15).

Finally, it is worth noting that the coexistence of superconductivity and ferromagnetism in BiS_2_-based superconductors has been observed, and the relation between charge-density wave (CWD) and superconductivity as well as weak antilocalization (WAL) effects have been discussed, as reported in the following.

**Figure 16:**
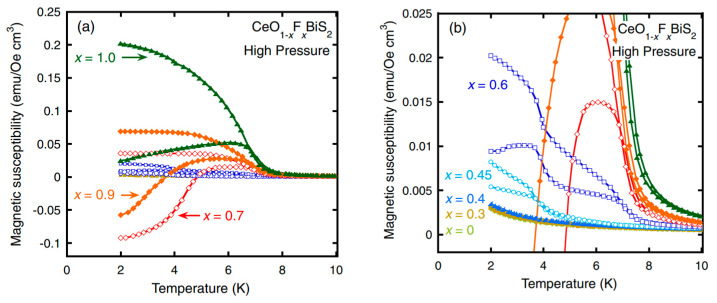
Temperature dependence of the magnetic susceptibility measured on CeO_1−x_F_x_BiS_2_ (for x = 0–1.0) after the high-pressure annealing. (**a**) Curves for x in the range from 0.7 to 1.0. (**b**) Curves for x in the range from 0 to 0.6. Figure is reproduced with permission from Ref. [192]. © (2014) The Physical Society of Japan.

CeO_1−x_F_x_BiS_2_ shows superconductivity after F substitution. Further, it simultaneously exhibits ferromagnetic-like ordering in the region where superconductivity exists [192,193]. After high-pressure annealing, superconductivity around 6 K appears when x = 0.7 and 0.9. In this region, ferromagnetism at 7.5 K is dominant. CeO_0.3_F_0.7_BiS_2_ shows the strongest diamagnetic signal among all the samples, while exhibiting ferromagnetic ordering below 7.5 K (see Figure 16).

YbO_1−x_F_x_BiS_2_ material also shows both superconductivity and antiferromagnetic-like ordering below 5.4 K (T_C_) [45]. In fact, the ZFC and FC magnetic susceptibility vs. T data for YbO_0.5_F_0.5_BiS_2_ both reveal changes and hysteresis at T_C_ and peaks at a temperature of about 2.7 K that appear to be due to magnetic ordering. This interpretation is further supported by a sharp feature in the specific heat near 2.7 K. The relation between CDW and superconductivity has been widely debated, as CDW turns out to be present as a ground state in the phase diagram of many unconventional superconductors, for example, cuprate or transition-metal dichalcogenides (TMDC) superconductors [194,195]. A long-range CDW ground state that coexists with superconductivity has been observed in NdO_0.6_3F_0.3_7BiS_2_ single crystals using high-energy and high-flux X-ray diffraction [196]. From the data, the authors deduce that the CDW is a bulk intrinsic property, rather than a surface reconstruction. They also suggest that CDW and superconductivity may coexist over a certain doping range in the phase diagram. Like lattice vibrations in a conventional superconductor, charge/orbital fluctuation near the quantum critical point could be capable of mediating superconductivity.

**Figure 17:**
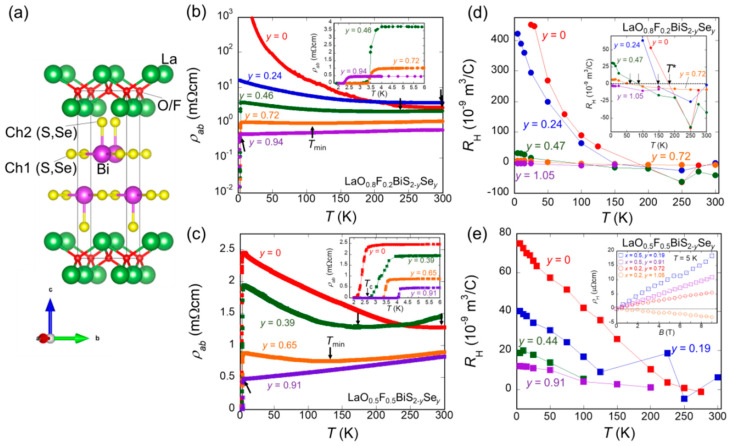
Electrical resistivity and Hall coefficients of LaO_1−x_F_x_BiS_2−y_Se_y_ (x = 0.2, 0.5, y = 0–1.05). (**a**) Schematic of the crystal structure (solid line indicates the unit cell). (**b**,**c**) Temperature dependence of resistivity in the ab-plane, for (**b**) x = 0.2 and (**c**) x = 0.5. Different colors indicate Se concentration. The arrows in (**b**,**c**) indicate the T value at which the resistivity above 5 K has a minimum. (**d**,**e**) Temperature dependence of the Hall coefficient for (**d**) x = 0.2 and (**e**) x = 0.5. The arrows in the inset of (**d**) indicate the sign-change temperature of the Hall coefficient. Reproduced from Ref. [197]. © (2014) The Physical Society of Japan.

The transport properties of layered LaO_1−x_F_x_BiS_2−y_Se_y_ (x = 0.2, 0.5, y = 0–1.05) have shown possible WAL [197]. Electrical resistivity and Hall coefficients (see Figure 17) present an increasing behavior with decreasing temperature, stronger for Se-poor samples. The moderately Se-substituted samples exhibit metallic behavior at high temperatures and a weak increase in the resistivity at low temperatures, suggesting the existence of carrier localization. The heavily Se-substituted compounds show metallic behavior in the whole temperature range; magnetoresistance measurements indicate that WAL is realized in these samples. A crossover state between WAL and weak localization (WL) emerges around the moderately F-doped and Se-free LaO_0.8_F_0.2_BiS_2_. The authors propose that the BiCh_2_-based system is a good candidate for studying the relationship between localization and superconductivity.

## 5. Conclusions

In this review, the numerous theoretical and experimental results concerning superconducting materials of the BiS_2_ family are summarized. These materials are generally insulators with a band gap. It is the electron doping of the BiS_2_ planes that makes the material metallic and induces superconductivity. One of the most interesting characteristics of these materials is the dependence of their superconducting properties on variations in the local crystal structure and stress. Indeed, it has been demonstrated that the application of high external pressure (or even sample preparation under high-pressure conditions) causes a significant increase in the critical temperature. The highest T_C_ (about 11 K) in this family of materials has been achieved in the high-pressure phase of LaO_0.5_F_0.5_BiS_2_. Although the mechanisms underlying superconductivity in BiS_2_-based materials have not yet been clarified, several important properties have been discovered. First, the anisotropy of superconductivity is extremely high, similar to cuprates. Additionally, large superconducting fluctuations have been observed in single crystals of NdO_0.5_F_0.5_BiS_2_ and polycrystalline samples of Bi_4_O_4_S_3_, suggesting that Cooper pairs could form at temperatures above the bulk T_C_. The role of charge density waves, the low charge carrier density, and the strong coupling are certainly topics of great interest and are under the attention of the community for future investigations to clarify the mechanism responsible for superconductivity in these materials.

## Figures and Tables

**Table 1:** List of some BiS_2_-based superconducting compounds with their critical temperature.

Material	T_C_ (K)	Ref.	Material	T_C_ (K)	Ref.
Bi_4_O_4_S_3_	6	[41,42,50,51]	La(O,F)BiS_2_	11.5	[43,52,53,54,55,56,57,58]
Bi_4_O_4_(S,Se)_3_	4.5	[59]	(La,Sm)(O,F)BiS_2_	10.5	[60,61]
Bi_3_O_2_S_3_	6	[62,63]	La(O,F)Bi(S,Se)_2_	4	[49,64,65,66]
CeOBiS_2_	4	[67,68]	La(O,F)BiSe_2_	6.5	[69,70,71]
LaOBiS_2_	3.5	[43,72,73,74]	Pr(O,F)BiS_2_	7	[75,76,77]
Bi(O,F)BiS_2_	5	[78,79,80]	Nd(O,F)BiS_2_	6.5	[81,82,83,84,85]
Ce(O,F)BiS_2_	8	[86,87,88,89]	Yb(O,F)BiS_2_	5	[45]
